# Impact of screening for breast cancer in high-risk women on health-related quality of life

**DOI:** 10.1038/sj.bjc.6601912

**Published:** 2004-06-15

**Authors:** A J Rijnsburger, M L Essink-Bot, S van Dooren, G J J M Borsboom, C Seynaeve, C C M Bartels, J G M Klijn, A Tibben, H J de Koning

**Affiliations:** 1 Department of Public Health, Erasmus MC, University Medical Center Rotterdam, PO Box 1738, 3000 DR Rotterdam, The Netherlands; 2 Department of Medical Psychology and Psychotherapy, Erasmus MC, University Medical Center Rotterdam, PO Box 1738, 3000 DR Rotterdam, The Netherlands; 3 Family Cancer Clinic, Department of Medical Oncology, Erasmus MC, Daniel den Hoed Cancer Center, PO Box 5201, 3008 AE Rotterdam, The Netherlands; 4 Department of Surgical Oncology, Erasmus MC, Daniel den Hoed Cancer Center, PO Box 5201, 3008 AE Rotterdam, The Netherlands; 5 Center of Human and Clinical Genetics, Leiden University Medical Center, PO Box 9600, 2300 RC Leiden, The Netherlands

**Keywords:** breast cancer, screening, quality of life, high risk

## Abstract

The effectiveness of intensive surveillance in women at high risk for breast cancer due to a familial or genetic predisposition is uncertain and is currently being evaluated in a Dutch magnetic resonance imaging (MRI) screening (MRISC) study, in which annual imaging consists of mammography and MRI. Unfavourable side effects on health-related quality of life may arise from this screening process. We examined the short-term effects of screening for breast cancer in high-risk women on generic health-related quality of life and distress. A total of 519 participants in the MRISC study were asked to complete generic health-status questionnaires (SF-36, EQ-5D) as well as additional questionnaires for distress and items relating to breast cancer screening, at three different time points around screening. The study population showed significantly better generic health-related quality of life scores compared to age-/sex-adjusted reference scores from the general population. Neither generic health-related quality of life scores nor distress scores among the study sample (*n*=334) showed significant changes over time. The impact of the screening process on generic health status did not differ between risk categories. Relatively more women reported mammography as quite to very painful (30.1%) compared to MRI. Anxiety was experienced by 37% of the women undergoing MRI. We conclude that screening for breast cancer in high-risk women does not have an unfavourable impact on short-term generic health-related quality of life and general distress. In this study, high-risk women who opted for regular breast cancer screening had a better health status than women from the general population.

Women in Western countries have a 8–10% average lifetime risk of developing breast cancer. One of the risk-increasing factors is a family history of breast cancer (Claus *et al*, 1991; Peto *et al*, 1996). About 5–10% of all breast cancer cases occur in women with a strong family history, and in the Netherlands approximately 25% of these cases may be attributed to the BRCA1 and BRCA2 breast cancer susceptibility gene mutations (Verhoog *et al*, 2001). Several strategies to reduce the risk of breast cancer or breast cancer death may be discussed with BRCA 1/2 mutation carriers and women with a strong family history, such as intensive surveillance, chemoprevention (Cuzick *et al*, 2003) and prophylactic mastectomy (Meijers-Heijboer *et al*, 2001). Guidelines for surveillance of these women mostly consist of biannual clinical breast examination (CBE), annual mammography and recommendation for monthly breast self-examination (BSE) (Vasen *et al*, 1998). Alternative imaging techniques like magnetic resonance imaging (MRI) may be useful because of reported high sensitivity in a diagnostic setting (Heywang-Kobrunner *et al*, 1997; Friedrich, 1998).

The effectiveness of intensive surveillance in women at high risk for breast cancer is yet uncertain, although preliminary results have been reported (Kuhl *et al*, 2000; Brekelmans *et al*, 2001; Stoutjesdijk *et al*, 2001; Warner *et al*, 2001; Kriege *et al*, 2003). It is currently being evaluated as part of a large ongoing prospective national MRI screening (MRISC) study in the Netherlands, in which annual imaging consists of mammography and MRI (Kriege *et al*, 2001). Unfavourable side effects on health-related quality of life (or health status) may arise from the process of screening itself, like pain, discomfort and feelings of anxiety and distress. Several studies have shown that women with normal results after mammography screening experience no important negative psychological consequences, whereas recall because of a false-positive mammogram causes adverse emotional, physical and social effects (Lerman *et al*, 1991; Cockburn *et al*, 1994; Sutton *et al*, 1995; Gilbert *et al*, 1998). Only one study reported that screening appeared to be less stressful for women with a family history than for those without (Gilbert *et al*, 1998).

This article describes the short-term effects of screening for breast cancer in high-risk women on health-related quality of life, by empirical assessment at various stages in the screening process. It addresses two specific questions: (1) Does the screening process have any impact, negative or positive, on generic health-related quality of life and distress among high-risk women? (2) Do high-risk women who opt for regular screening differ from the general population with respect to generic health-related quality of life?

## MATERIALS AND METHODS

### MRISC study

The MRISC study, activated at six family cancer clinics in the Netherlands, is an ongoing prospective observational study for women at increased risk for breast cancer due to a familial or genetic predisposition (Kriege *et al*, 2001). The study was designed to investigate the effectiveness of intensive surveillance and the value of MRI compared to mammography as a screening tool in high-risk women. Women who were already under intensive surveillance and women who came for the first time to the family cancer clinic were asked to participate in the MRISC study. Women with evident symptoms suspicious for breast cancer or previous breast cancer were excluded. Participants visited the family cancer clinic twice a year for surveillance, consisting of biannual CBE and annual mammography and MRI. All women got instructions for monthly BSE. Since the start of the study in 1999, 1952 women have been included. The first results were recently presented (Kriege *et al*, 2003). Approval for the MRISC study was obtained from the Medical Ethical Committees of all six participating family cancer clinics. The health-related quality of life study was approved by the Medical Ethical Committee of the Erasmus MC, University Medical Center Rotterdam.

### Design of the study on health-status effects of screening high-risk women

Participants in the MRISC study who were under surveillance at the Family Cancer Clinic of the Erasmus MC, Daniel den Hoed Cancer Center were approached for the empirical health-status study either by mail or by their physician at a scheduled visit at the family cancer clinic. Women received written information about the study, including an informed consent form and a form on which they could indicate that they did not want to participate. Women could send the appropriate form back in a reply paid envelope. A reminder letter was sent to those women who did not return any form within 4 months.

**Figure 1:**
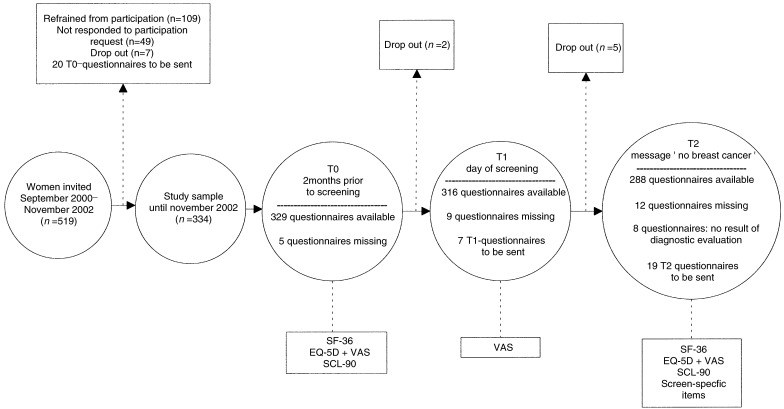
Flow chart describing the number of questionnaires available for statistical analysis (cutoff point November 2002).

Health-status data were collected at the time points outlined in Figure 1

. At 2 months prior to the scheduled screening visit (consisting of either CBE alone or CBE in combination with mammography and MRI) participating women received their first (baseline) questionnaire (time 0 or T0) by mail. They were requested to fill it in and send it back within 2 weeks. The second assessment (time 1 or T1) took place at the day of the scheduled screening visit, preceding the screening. Postscreening measurement (time 2 or T2) was performed 1 week (in case of CBE alone) or 4 weeks (in case of CBE in combination with mammography and MRI) after screening. By that time all women had been informed whether they had breast cancer or not, including those who received additional diagnostic evaluation after scheduled screening. Women with a screen-detected or interval breast cancer did not receive any questionnaire after the diagnosis. Women who did not return their questionnaire within 4 weeks were sent a reminder.

### Health-status measures

Health-related quality of life was defined as the woman's functioning in physical, psychological and social domains. The questionnaire contained the Medical Outcomes Study 36-Item Short Form (SF-36) (Ware and Sherbourne, 1992; Ware *et al*, 1994; Aaronson *et al*, 1998) as a generic health profile measure and the EQ-5D as a generic preference-based measure of health-related quality of life (Brooks, 1996; Dolan, 1997). In addition it contained the somatic subscale (SOM) of the Symptom Checklist-90 (SCL-90) (Derogatis, 1977), self-developed screen-specific items (Essink-Bot *et al*, 1998) and other measures (to be reported elsewhere). We used both the SOM scale and the role-emotional and mental health scales of the SF-36 as measures for distress. In the screen-specific items, women were retrospectively (at T2) asked to grade the pain, discomfort and anxiety experienced during CBE, mammography and MRI. For further details on the health-status measures, we refer to Appendix A.

Finally, the questionnaire contained items on sociodemographic characteristics (including age, marital status, living status (single or together), parity, educational level and employment status) and cancer-related characteristics (the number of years adhering to regular surveillance, frequency of BSE, benign breast symptoms in the past, diagnosis of different type(s) of cancer in the past and family history (mother and/or sister(s) affected with breast cancer)).

### Subgroups

Participating women were divided into subgroups according to three criteria. The first was their cumulative lifetime risk (CLTR) for developing breast cancer, based on the tables of Claus (Claus *et al*, 1994) and additional information about the family history of ovarian cancer (Kriege *et al*, 2001). Risk category 1 consisted of BRCA 1/2 mutation carriers (50–85% CLTR), category 2 being women with high risk for breast cancer (30–50% CLTR) and category 3 comprising women with moderate risk for breast cancer (15–30% CLTR). We also distinguished subgroups according to screening modality (two subgroups: CBE alone or CBE in combination with mammography and MRI) and additional diagnostic evaluation after the scheduled screening (two subgroups: yes or no).

### Statistical analysis

Missing values for the SF-36 items were imputed according to the standard guidelines (Ware *et al*, 1993). No imputation of missing values was applied to the rest of the variables.

Differences in distribution of background variables between the different subgroups were analysed by means of the *χ*^2^ test, Fisher's exact test or linear-by-linear association (nominal and ordinal variables), Student's *t*-test or ANOVA (continuous variables with normal distribution) and by nonparametric procedures (continuous variables without normal distribution: Mann–Whitney or Kruskal–Wallis test). Age- and sex-adjusted reference scores for the SF-36 and EQ-5D were assigned to participating women at T0, based on their age at T0. The one-sample *t*-test was used to test whether the difference between the reference and the observed health-related quality of life scores differed systematically from zero. As only sex- and no age-adjusted reference scores for the SOM scale were available, we analysed the differences between observed SOM scale scores and sex-adjusted reference scores by means of a *t*-test for two independent samples allowing for unequal variances.

To evaluate changes over time in generic health-related quality of life scores (SF-36 and EQ-5D) and in SOM scale scores for the total group of women, we used a repeated measures ANOVA model with time as the only main effect. Differences in health-related quality of life and SOM scale scores between the various subgroups, including differences over time, were also examined with repeated measures ANOVA models. Three models were fitted, each including the main effect for time, and one of the three factors: risk category (model A), screening modality (model B) and additional diagnostic evaluation (model C); all models included the interaction effect for time with one of the three factors. For each model, selection of relevant confounders was done by initially including age and those background variables (Table 1

**Table 1:** Baseline characteristics of participating women at T0 according to risk category, screening modality and additional diagnostic evaluation after screening

		**Risk category (*n*=329)**			**Screening modality (*n*=329)**		**Additional evaluation (*n*=288)**	
	**Total (*n*=329)**	**50–85% (*n*=35)**	**30–50% (*n*=186)**	**15**–**30% (*n*=108)**	**CBE (*n*=163)**	**CBE+mammography +MRI (*n*=166)**	**Yes (*n*=21)**†	**No (*n*=267)**
*Age (years)*								
Mean (s.d.)	40.9 (8.9)	41.3 (11.0)	41.4 (8.8)	40.0 (8.4)	41.1 (9.3)	40.8 (8.6)	44.5 (8.7)	40.9 (8.9)‡
								
*Marital status (%)*								
Married/registered partnership	75.5	77.1	77.7	71.3	75.2	75.9	76.2	75.4
Never married	16.8	22.9	13.6	20.4	14.9	18.7	9.5	17.0
Divorced/widowed	7.6	0	8.7	8.3	9.9	5.4	14.3	7.6
								
*Living status (%)*								
Living single	10.3	11.8	8.2	13.5	10.6	10.0	14.3	10.9
Living together	89.7	88.2	91.8	86.5	89.4	90.0	85.7	89.1
								
*Parity (%)*								
Yes	71.7	60.0	74.7	70.4	70.6	72.9	85.7	70.7
No	28.3	40.0	25.3	29.6	29.4	27.1	14.3	29.3
								
*Educational level (%)*								
Low	37.0	45.2	37.6	33.3	45.6	28.7*	31.6	36.6
Intermediate	35.0	25.8	34.1	39.6	31.3	38.7	31.6	36.1
High	27.9	29.0	28.2	27.1	23.1	32.7	36.8	27.3
								
*Employment status: paid job (%)*								
Yes	73.7	65.7	74.1	75.7	74.1	73.3	66.7	75.4
No	26.3	34.3	25.9	24.3	25.9	26.7	33.3	24.6
								
*Number of years adhering to regular surveillance*								
Mean (s.d)	5.4 (4.6)	3.0 (1.7)	5.6 (4.6)	5.8 (5.1)*	5.9 (5.1)	4.9 (4.1)‡	7.3 (6.0)	5.3 (4.7)
								
*Frequency of breast self-examination (%)*								
At least once a week	13.5	35.3	10.4	12.0*	12.5	14.5	28.6	11.5
Approximately once a month	57.2	58.8	58.5	54.6	57.5	57.0	52.4	57.6
Approximately once every 3/6/12 months	19.4	5.9	21.9	19.4	18.1	20.6	9.5	21.0
Never	9.8	0	9.3	13.9	11.9	7.9	9.5	9.9
								
*Benign breast diseases in the past (%)*								
Yes	38.8	14.3	41.8	41.7*	36.0	41.6	71.4	36.0*
No	61.2	85.7	58.2	58.3	64.0	58.4	28.6	64.0
								
*Diagnosis of different type(s) of cancer in the past (%)*								
Yes	4.0	5.7	4.3	2.8	3.7	4.2	4.8	4.2
No	96.0	94.3	95.7	97.2	96.3	95.8	95.2	95.8
								
*Mother and/or sister(s) affected with breast cancer (%)*								
Yes	88.6	69.7	93.5	86.0*	93.2	84.1*	95.2	87.8
No	11.4	30.3	6.5	14.0	6.8	15.9	4.8	12.2

CBE=clinical breast examination; MRI=magnetic resonance imaging.

* *P*-value ⩽0.01.

† Eight women with additional diagnostic evaluation were excluded from the analyses, because they were still waiting for the results.

‡ *P*-value ⩽0.10.

) with a significant (*P*⩽0.10) difference in distribution between the relevant subgroups as covariates in the model, and then by removing covariates that did not show a significant (*P*⩽0.05) confounding effect on any of the outcome scores between the subgroups. In all models, we used a compound symmetry covariance structure. The parameters of these covariance matrices were allowed to differ between groups in the models that included one of the three group factors, as this provided for better fitting models.

All *P*-values resulted from the use of two-sided statistical tests. The data analyses were performed using SPSS (SPSS 10.0.7 for Windows; SPSS Inc., Chicago, IL, USA) or the MIXED procedure of SAS (SAS 8.00 TS Level 00M0 for Windows; SAS Institute Inc., Cary, NC, USA).

## RESULTS

### Characteristics of the study group

From September 2000 to November 2002, 519 women were approached for the health-status study; 69.6% consented to participate (Figure 1). At November 2002, we had 329 (T0), 316 (T1) and 288 (T2) completed and evaluable questionnaires. Response rates were high among those who received a questionnaire (T0: 98.5%; T1: 96.6%; T2: 94.4%).

Sociodemographic and cancer-related background characteristics of the total study sample and the different subgroups are given in Table 1. The mean age at entry in the study was 40.9 years; 72% of the women had a low to intermediate level of education. The mean number of years already adhering to regular surveillance was 5.4 years, but this differed significantly (*P*⩽0.01) between the three risk categories. Of the total study sample, 1.6% just started with regular surveillance. Most of the women (88.6%) had (had) a mother and/or sister(s) affected with breast cancer.

No significant differences with regard to age (*P*=0.38) and CLTR of developing breast cancer (*P*=0.36) were found between the study sample (*n*=334) and the women who refrained from participation in the health-status study (*n*=109). There were also no significant differences with regard to sociodemographic and cancer-related background characteristics and baseline SF-36, EQ-5D and SOM scale scores between the 288 women with a usable T2 questionnaire and the 46 women (334–288) without a usable T2 questionnaire, except for the vitality score of the SF-36, which was lower for the women without a usable T2 questionnaire (68.1 *vs* 60.3, *P*⩽0.05).

### Health-related quality of life over time

The mean score results from the SF-36, EQ-5D and SOM scale at different time points around screening (T0, T1 and T2) are shown in Table 2

**Table 2:** Observed SF-36, EQ-5D and SOM scale (SCL-90) scores (mean values and 25^th^–75th percentile score intervals) of participating women at T0, T1 and T2; comparison with (age-/sex-adjusted) reference scores

	**T0 (*n*=329): mean (25th–75th percentile)**	**T1 (*n*=316): mean (25th–75th percentile)**	**T2 (*n*=288): mean (25th–75th percentile)**	**Reference scores SF-36: Dutch general population^a^**	**Reference scores SF-36: USA general population^b^**	**Reference scores EQ-5D^c^ and SOM scale^d^**
*SF-36 (score 100–0)*						
Physical functioning	89.9 (85.0–100.0)	—	89.4 (85.0–100.0)	86.3*	86.1*	
Role – physical	85.7 (100.0–100.0)	—	84.1 (100.0–100.0)	77.6*	82.8	
Bodily pain	82.4 (72.0–100.0)	—	83.0 (72.0–100.0)	72.8*	75.0*	
General health perceptions	76.4 (67.0–92.0)	—	77.3 (67.0–92.0)	72.2*	72.7*	
Vitality	67.1 (55.0–80.0)	—	68.9 (55.0–80.0)	64.8	59.3*	
Social functioning	87.7 (75.0–100.0)	—	87.9 (75.0–100.0)	83.5*	83.0*	
Role – emotional	85.2 (100.0–100.0)	—	88.1 (100.0–100.0)	80.1†	81.2†	
Mental health	76.8 (68.0–88.0)	—	77.7 (68.0–88.0)	74.4†	73.4*	
						
*SF-36 summary scores*						
Physical component summary	52.5 (49.8–57.7)	—	52.3 (49.2–57.7)	50.0*	50.7*	
Mental component summary	51.2 (48.3–57.8)	—	52.2 (48.6–58.0)	50.1	49.1*	
						
*EQ-5D*						
Utility score (score 1–0)	0.88 (0.80–1.00)	—	0.88 (0.80–1.00)			0.85*
VAS (self-rated health today) (score 100–0)	81.9 (73.0–90.0)	79.0 (70.0–90.0)	80.7 (70.0-90.0)			86.9*
						
SOM scale SCL-90 (score 12–60)	17.5 (14.0–19.0)	—	17.1 (13.0–19.0)			18.7‡

SOM=somatic subscale; SCL-90=Symptom Checklist-90; SF-36=Medical Outcomes Study 36-Item Short Form; VAS=visual analogue scale.

a Age- and sex-adjusted SF-36 reference scores (women of 16–65 years of age) from the Dutch general population (Aaronson *et al*, 1998). Scores were assigned to the sample of participating women at T0, based on their age at T0.

b Age- and sex-adjusted SF-36 reference scores (women of 18–64 years of age) from the USA general population (Ware *et al*, 1993, 1994). Scores were assigned to the sample of participating women at T0, based on their age at T0.

c Age- and sex-adjusted EQ-5D reference scores (women of 20–69 years of age) from the Swedish general population (Burström, 2003). Scores were assigned to the sample of participating women at T0, based on their age at T0.

d Sex-adjusted SOM scale reference scores (women of 18–83 years of age) from the Dutch general population (Arrindell and Ettema, 1986).

* Statistically significant (*P*-value ⩽0.01) difference between observed scores of participating women at T0 and age-and sex-adjusted reference scores (one sample *t*-test with best value 0).

† Statistically significant (*P* value ⩽0.05) difference between observed scores of participating women at T0 and age- and sex-adjusted reference scores (one-sample *t*-test with test value 0).

‡ Statistically significant (*P*-value ⩽0.01) difference between observed scores of participating women at T0 and sex-adjusted reference scores (two independent samples *t*-test with unequal variances).

. For the total group of women, there was a significant (*P*⩽0.01) but small change in visual analogue scale (VAS) scores over time. A *post hoc* analysis revealed that the mean VAS score at T0 (81.9) differed significantly (*P*⩽0.01) from the mean T1 score (79.0), which in itself differed significantly (*P*⩽0.05) from the mean T2 score (80.7). All other generic health-related quality of life scores (SF-36 and EQ-5D utility) and SOM scale scores did not show any significant change over time.

High-risk women showed significantly (*P*⩽0.01/*P*⩽0.05) higher SF-36 scores on most scales as compared to the age- and sex-adjusted SF-36 reference scores from the Dutch (Aaronson *et al*, 1998) and USA (Ware *et al*, 1993; 1994) general population (Table 2). Also, observed EQ-5D utilities and SOM scale scores among our study sample were significantly (*P*⩽0.01) more favourable compared to the age- and sex-adjusted EQ-5D utility reference scores from the Swedish general population (Burström, 2003) and sex-adjusted SOM scale reference scores from the Dutch general population (Arrindell and Ettema, 1986). Observed VAS scores among our study sample were significantly lower (*P*⩽0.01) compared to the age- and sex-adjusted reference scores from the Swedish general population (Burström, 2003).

### Differences in health-related quality of life between subgroups over time

Covariates included in the final repeated measures ANOVA models were age (models A, B and C), educational level (model B), number of years adhering to regular surveillance (models A and B), frequency of BSE (model A) and mother and/or sister(s) affected with breast cancer (models A and B).

The analyses with model A revealed no significant interaction between risk category and time (*P*-value range 0.16–0.73) for any of the SF-36, EQ-5D and SOM scales. For none of these scales there was a significant main effect of risk category (*P*-value range 0.12–1.00). The same holds for model B: there were no significant interaction effects between screening modality and time (*P*-value range 0.15–0.95), and no significant main effect of screening modality (*P*-value range 0.12–1.00) for any of the outcome scales. Only for the VAS, there was a significant (*P*⩽0.01) interaction between additional diagnostic evaluation and time (model C). Women without additional diagnostic evaluation after scheduled screening had higher VAS scores at baseline than those undergoing additional diagnostic procedures (83.0 *vs* 72.4, *P*⩽0.01), but this difference disappeared at T1 and T2. For the other scales, no significant interaction effects between additional diagnostic evaluation and time (*P*-value range 0.053–0.87) were seen. There was no significant main effect of additional diagnostic evaluation (*P*-value range 0.13–0.96) for any of the outcome scales.

### Pain, discomfort and anxiety during different screening modalities

Of the women who underwent a screening mammography, 21.1% described pain intensity as ‘quite’ and 9.0% as ‘very’ (Table 3

**Table 3:** Pain, discomfort and anxiety experienced during relevant screening tests, as reported by participating women in the T2 questionnaire (*n*=288: 147 women CBE, 141 women CBE+mammography+MRI)

	**CBE** **(*n*=287)^a^**	**Mammography (*n*=134)^a^**	**MRI** **(*n*=109)^a^**
*Pain (%)*			
Not	92.6	14.3	88.0
A little	6.7	55.6	11.1
Quite	0.7	21.1	0.9
Very	0	9.0	0
			
*Discomfort (%)*			
Not	91.5	30.8	54.6
A little	7.4	47.4	36.1
Quite	0.7	15.8	4.6
Very	0.4	6.0	4.6
			
*Anxiety (%)*			
Not	77.9	72.4	63.0
A little	20.4	22.4	26.9
Quite	1.4	4.5	7.4
Very	0.4	0.7	2.8

CBE=clinical breast examination; MRI=magnetic resonance imaging.

a One of 288 women reported no CBE during last screening; seven of 141 women reported no mammography during last screening; 32 of 141 women reported no MRI during last screening.

). Also 12% of the women reported the MRI to be painful. A large proportion of the women experienced any discomfort during mammography (69.2%) and MRI (45.3%). Anxiety was mainly experienced during MRI, with 10.2% of the total describing anxiety intensity as ‘quite’ to ‘very’.

## DISCUSSION

Intensive surveillance in women at high risk for breast cancer due to a familial or genetic predisposition is increasing. As its effectiveness is yet uncertain, it is important to pay attention to possible unfavourable side effects on health-related quality of life and distress, which may arise from the screening process. As far as we know, no studies had been performed before to investigate the short-term effects of intensive surveillance on health-related quality of life in high-risk women. This study showed that screening women at increased risk for breast cancer, as performed in this study by biannual CBE and annual mammography and MRI, did not have a relevant impact on generic health-related quality of life. Most of the women in our study underwent MRI for the first time.

The results do not provide evidence for a distress-raising effect of screening. The mean SOM scale scores and role-emotional and mental health scores of the SF-36 at 2 months prior and 1–4 weeks after screening did not show any significant difference. There are several possible explanations for this result. First, various coping processes can generate and sustain positive psychological states in the context of highly stressful circumstances, thereby minimizing or avoiding the adverse mental and physical health effects of distress (Folkman, 1997). Second, opting for regular screening may give women the feeling that they do everything they can to handle their risk of getting breast cancer, eliminating possible distress-raising effects of screening. Third, the SF-36 was not administered at the day of the screening tests, when distress levels could be elevated. Instead, pre- and postscreening measurement took place 2 months before and 1–4 weeks after screening. Fourth, most of the women adhered to regular surveillance already for a longer time, which may have lowered distress levels. A fifth explanation relates to the method of measuring distress using the SOM scale and the role-emotional and mental health scale of the SF-36, which may be too general. The use of a specific measure of psychological consequences of breast cancer screening could provide additional insight (Cockburn *et al*, 1992; 1994).

Interestingly, it appeared that our study population showed significantly better generic health-related quality of life scores (SF-36, EQ-5D utility and SOM scale) as compared to the age-/sex-adjusted reference scores. It seems that high-risk women who choose for regular screening have a better health status than women from the general population. Most of the women opt for intensive surveillance voluntarily, and this may result in a selection of healthy and well-coping women. This was also seen in the Rotterdam screening trial for prostate cancer, where health status among the voluntary attenders was better than among the general population (Essink-Bot *et al*, 1998). The difference in health status between our study sample and the general population may partly be due to difference in educational level. Participants in the health-status study appeared to have a significant (*P*=0.03) higher educational level compared to Dutch women aged 15–64 years (Statistics Netherlands, 2004). Individuals with a higher education are more likely to undergo screening (Liu *et al*, 2001). Moreover, higher levels of education are also associated with higher levels of quality of life (Regidor *et al*, 1999).

Women who refrained from participation in the health-status study all opted for intensive surveillance. They did not differ from our study sample with respect to age and risk category. Nevertheless, it is still possible that they differed from our study sample with respect to health-related quality of life, but we had no data available.

Observed VAS scores among our study sample were significantly lower compared to the age- and sex-adjusted reference scores. This may be caused by the fact that the labelled anchors of the Swedish reference scores were ‘dead’ and ‘full health’, instead of ‘worst imaginable health state’ and ‘best imaginable health state’ (Burström, 2003).

The VAS score was the only generic health-related quality of life score that showed a significant change over time. However, the absolute differences between the mean VAS scores were small.

Generic health-related quality of life, as well as the impact of the screening process on generic health-related quality of life, did not differ between the three risk categories. These risk categories represent the baseline objective CLTR of developing breast cancer, based on the tables of Claus (Claus *et al*, 1994). In these analyses, we did not take into account the women's cognitive or affective perceptions of their risk of developing breast cancer.

Relatively more women reported mammography as quite to very painful (30.1%) compared to CBE or MRI, while a large proportion of the women experienced any discomfort during mammography (69.2%) and MRI (45.3%). The documented incidence of pain associated with screening mammography varies from 1 to 62% (Sapir *et al*, 2003). Patient education by trained nursing counsellors may reduce mammography-related pain and discomfort (Nielsen *et al*, 1993). Since all mammograms in our study sample were performed at the Erasmus MC, Daniel den Hoed Cancer Center by extremely skilled technicians who inform and support the women, and the majority of the women did not undergo mammography for the first time (contrary to MRI), we think that a lack of information with respect to pain and discomfort experience at mammography is not the issue, rather than the examination itself.

Anxiety was experienced by 37% of the women undergoing MRI. Of the women participating in the MRISC study, 1.8% stopped the study protocol because they refused another MRI or were anxious for the MRI exam (unpublished data).

We did not investigate the impact of additional diagnostic work-up on generic health-related quality of life during recall. There is evidence that recall after a false-positive mammogram causes elevated levels of anxiety and breast cancer worries, even after receiving reassurance that all is well (Lerman *et al*, 1991; Cockburn *et al*, 1994). More research on this item is warranted.

Besides intensive surveillance, prophylactic mastectomy is an alternative risk reducing strategy, especially for BRCA 1/2 mutation carriers (Meijers-Heijboer *et al*, 2001). In the Family Cancer Clinic of the Erasmus MC, approximately half of the unaffected BRCA 1/2 mutation carriers opts for prophylactic mastectomy (Meijers-Heijboer *et al*, 2000). However, the use and accessibility of prophylactic surgery differs largely between countries (Julian-Reynier *et al*, 2001). Results from studies on the impact of prophylactic mastectomy on generic health-related quality of life are not available in the literature. Utility ratings of prophylactic oophorectomy and mastectomy seem low, although reduction in anxiety was not taken into account (Grann *et al*, 1998). Lodder *et al* (2002) showed that women opting for prophylactic mastectomy had significant higher distress levels than mutation carriers who opted for surveillance, but their distress levels decreased significantly 6 months or longer after surgery, possibly due to the significant risk reduction of developing breast cancer.

We conclude that the screening process does not have an unfavourable impact on short-term generic health-related quality of life and general distress in women at high risk for breast cancer. In this study, high-risk women who opted for regular breast cancer screening had a better health status than women from the general population, which may partly be due to difference in educational level.

## Appendix A

### HEALTH-STATUS MEASURES

The SF-36 (Ware and Sherbourne, 1992) includes eight multiitem scales: physical functioning, role limitations due to physical problems (role-physical), bodily pain, general health perceptions, vitality, social functioning, role limitations due to emotional problems (role-emotional) and mental health. It produces a health profile with scores between 0 and 100 for each dimension, with higher scores indicating a better health status (Ware *et al*, 1993). Two summary scale scores can be computed that aggregate the eight scales: the physical component summary and the mental component summary. These summary scores have a mean score of 50 (s.d.=10) for the general United States population (Ware *et al*, 1994). We used a validated Dutch version of the SF-36 (Aaronson *et al*, 1992; 1998) with a time frame (reference period) of 1 week.

The EQ-5D (Brooks, 1996) is a five-item self-classifier with regard to mobility, self-care, usual activities, pain/discomfort and anxiety/depression. Each item has three levels: no problems (1), some problems (2) and extreme problems (3). The derived health state descriptions can be linked directly to empirical valuations of health states by the general population, generating utilities that can be used in the calculation of quality adjusted life years (Dolan, 1997). The EQ-5D also contains a VAS on which respondents rate their own health between 0 (labelled as ‘worst imaginable health state’) to 100 (labelled as ‘best imaginable health state’). For this study, the standard Dutch EQ-5D was used (EuroQol, 1996).

The SCL-90 (Derogatis *et al*, 1973; Derogatis, 1977) is a self-report symptom inventory for the measurement of psychological distress and psychopathology. The 12-item SOM scale measures complaints about general physical dysfunction as a result of psychogenic or stress-related problems. However, the possibility of actual physical disabilities has to be taken into account. Each item has five levels: not at all (1), a little (2), quite (3), very (4) and extremely (5), providing a total scale score between 12 and 60.

The screen-specific items are self-developed items on experiences during the different screening tests. They are based on items developed to grade pain and physical discomfort of various prostate cancer screening tests (Essink-Bot *et al*, 1998). Women were retrospectively (at T2) asked to grade pain, discomfort and anxiety, experienced during CBE, mammography and MRI, respectively. Each item has answer categories on a 4-point Likert scale with labelled end points ‘not’ and ‘very’.
